# Supplementary material for: Delivering maternal and childcare at primary healthcare level: The role of PMAQ as a pay for performance strategy in Brazil
Source: PLoS One. 2020 Oct 15;15(10):e0240631. doi: 10.1371/journal.pone.0240631 (PMC7561084; doi:10.1371/journal.pone.0240631)
Supplement: S1 Table — (DOCX) [file pone.0240631.s001.docx]

**Table S1**. Description of variables used in the analysis

| **Variable** | **Definition** | **Data availability** | **Measure** | **Source** |
| --- | --- | --- | --- | --- |
| Outcomes – process indicator | | | | |
| Average number of antenatal consultations per month | Total number of antenatal consultations divided by total number of pregnant registered at primary healthcare team per month | Data reported monthly by primary healthcare team | Average of the period analysed | Information System for Primary Healthcare (SIAB, Portuguese acronym) |
| Average number of consultations for childcare under 2 years old per month | Total number of physician or nurse consultations for children under 2 years divided by the total number of children under 2 years registered at primary healthcare team per month | Data reported monthly by primary healthcare team | Average of the period analysed | Information System for Primary Healthcare (SIAB, Portuguese acronym) |
| Average number of physician consultations for children under 1 year old per month | Total number of physician consultations for children under 1 year divided by the total number of children registered at primary healthcare team per month | Data reported monthly by primary healthcare team | Average of the period analysed | Information System for Primary Healthcare (SIAB, Portuguese acronym) |
| Characteristics of family health team | | | | |
| Dummy for primary healthcare team with dental care | Besides regular health professionals (physician, nurse, nurse assistant and community health agent), primary healthcare team with dental care include dentist and dentist assistant | Data reported monthly by primary healthcare team | Dummy = 1 if more than 50% of period the team reported having dental care  Dummy = 0, if otherwise | National Register of Health  Establishments from  DATASUS (CNES/SUS) |
| Dummy for primary healthcare team with Family Health Support Team (NASF, acronym in Portuguese) | Besides regular health professionals (physician, nurse, nurse assistant and community health agent), primary healthcare team with NASF can include nutritionist, physical educator, physiotherapist, phycologist or others | Data reported monthly by primary healthcare team | Dummy = 1 if more than 50% of period the team reported having NASF  Dummy = 0, if otherwise | National Register of Health  Establishments from  DATASUS (CNES/SUS) |
| Total number of nurse working hours per month | Sum of working hours from all nurses registered in the primary healthcare team | Data reported monthly by primary healthcare team | Median of the period analysed | National Register of Health  Establishments from  DATASUS (CNES/SUS) |
| Total number of physician working hours per month | Sum of working hours from all physicians registered in the primary healthcare team | Data reported monthly by primary healthcare team | Median of the period analysed | National Register of Health  Establishments from  DATASUS (CNES/SUS) |
| Total number of dentist working hours per month | Sum of working hours from all dentists registered in the primary healthcare team | Data reported monthly by primary healthcare team | Median of the period analysed | National Register of Health  Establishments from  DATASUS (CNES/SUS) |
| Total number of nurse assistant working hours per month | Sum of working hours from all nurse assistants registered in the primary healthcare team | Data reported monthly by primary healthcare team | Median of the period analysed | National Register of Health  Establishments from  DATASUS (CNES/SUS) |
| Total number of dentist assistant working hours per month | Sum of working hours from all dentist assistants registered in the primary healthcare team | Data reported monthly by primary healthcare team | Median of the period analysed | National Register of Health  Establishments from  DATASUS (CNES/SUS) |
| Total number of community health agent working hours per month | Sum of working hours from all community health agents registered in the primary healthcare team | Data reported monthly by primary healthcare team | Median of the period analysed | National Register of Health  Establishments from  DATASUS (CNES/SUS) |
| Socioeconomic status | | | | |
| Socioeconomic status of the municipality where the primary healthcare team is in | Socioeconomic status is an index composed by GDP per capita, percentage of population with private health insurance, percentage of population with conditional cash transfer, percentage of population under extreme poverty, population density | Calculated by the Ministry of Health at the beginning of the PMAQ | Socioeconomic status was divided into six groups. Group 1 = poorest municipalities and Group 6 = the richest municipalities  (reference dummy) | Information System for Primary Healthcare (SIAB, Portuguese acronym) |
| Geographic characteristics | | | | |
| Dummy for rural area | When the primary healthcare team is in a rural area | Data reported monthly by primary healthcare team | Dummy = 1 if the primary healthcare team is in a rural area  Dummy = 0, if otherwise | National Register of Health  Establishments from  DATASUS (CNES/SUS) |
| Brazilian geographic region | Brazil is divided in five geographic regions (North, Northeast, Midwest, Southeast and South) | This variable do not change with time. | We set a dummy for each region. Northeast is defined as the reference case | Brazilian Institute of  Geography and Statistics (IBGE) |
| Population size of municipality | Population of municipalities were stratified by three strata: small (under 10,000 inhabitants), medium (from 10,001 to 100,000); and large (above 100,000) | Data reported annually by municipality | We set a dummy for each population size strata. Large population is defined as the reference case | Brazilian Institute of  Geography and Statistics (IBGE) |
