# Supplementary material for: Delivering maternal and childcare at primary healthcare level: The role of PMAQ as a pay for performance strategy in Brazil
Source: PLoS One. 2020 Oct 15;15(10):e0240631. doi: 10.1371/journal.pone.0240631 (PMC7561084; doi:10.1371/journal.pone.0240631)
Supplement: S2 Table — (DOCX) [file pone.0240631.s002.docx]

Table S2. Results from the OLS and QR models for antenatal consultations in the 1^st^ Cycle of PMAQ, Brazil

| Variable | PMAQ Cycle 1 | | | | | |
| --- | --- | --- | --- | --- | --- | --- |
|  | OLS | 10^th^ | 25^th^ | 50^th^ | 75^th^ | 90^th^ |
| PMAQ participating | .0566*** | .0809*** | .0860*** | .0521*** | .0281*** | .0098 |
|  | (.0045) | (.0080) | (.0073) | (.0055) | (.0053) | (.0062) |
| With dental care | -.0026 | .0141 | .0112 | .0039 | -.0036 | -.0240** |
|  | (.0085) | (.0148) | (.0134) | (.0101) | (.0098) | (.0113) |
| With NASF | .0251*** | .0675*** | .0378*** | .0194*** | .0084 | -.0081 |
|  | (.0045) | (.0083) | (.0076) | (.0058) | (.0056) | (.0064) |
| Characteristic of the team |  |  |  |  |  |  |
| hPhysician | .0005 | -.0019*** | -.0006 | .0017*** | .0028*** | .0031*** |
|  | (.0004) | (.0006) | (.0006) | (.0004) | (.0004) | (.0005) |
| hNurse | .0006 | .0013 | .0009 | .0014** | .0004 | .0007 |
|  | (.0005) | (.0009) | (.0008) | (.0006) | (.0006) | (.0007) |
| hDentist | .0001 | -.0005 | -.0004 | -.0000 | -.0001 | .0002 |
|  | (.0003) | (.0005) | (.0005) | (.0007) | (.0004) | (.0004) |
| hNurse assistant | .0014*** | .0018*** | .0019*** | .0013*** | .0011*** | .0008*** |
|  | (.0001) | (.0002) | (.0001) | (.0001) | (.0001) | (.0001) |
| hDentist assistant | .0005** | .0010*** | .0007** | .0006** | .0007*** | .0005* |
|  | (.0002) | (.0004) | (.0003) | (.0003) | (.0002) | (.0003) |
| hCommunity Health Agents | -.0003*** | -.0003*** | -.0005*** | -.0004*** | -.0003*** | -.0002*** |
|  | (.0000) | (.0000) | (.0000) | (.0000) | (.0000) | (.0000) |
| Socioeconomic status |  |  |  |  |  |  |
| Group 1 | .1831*** | .1353*** | .2155*** | .2001*** | .2072*** | .1752*** |
|  | (.0226) | (.0348) | (.0317) | (.0241) | (.0233) | (.0268) |
| Group 2 | .1078*** | .0956*** | .0967*** | .1339*** | .1456*** | .1141*** |
|  | (.0155) | (.0250) | (.0228) | (.0173) | (.0167) | (.0193) |
| Group 3 | .1060*** | .0724*** | .0762*** | .1282*** | .1469*** | .1248*** |
|  | (.0153) | (.0249) | (.0227) | (.0172) | (.0166) | (.0199) |
| Group 4 | .0687*** | .0505** | .0540** | .0932*** | .1199*** | .0963*** |
|  | (.0147) | (.0232) | (.0212) | (.0160) | (.0155) | (.0179) |
| Group 5 | .0167** | -.0001 | .0152 | .0334*** | .0361*** | .0071 |
|  | (.0081) | (.0140) | (.0128) | (.0097) | (.0094) | (.0108) |
| Geographic status |  |  |  |  |  |  |
| Rural area | -.0515*** | -.0560*** | -.0571*** | -.0509*** | -.0566*** | -.0443*** |
|  | (.0051) | (.0093) | (.0085) | (.0065) | (.0063) | (.0072) |
| North | -.0334*** | -.1486*** | -.0607*** | -.01707 | .01387 | .05033*** |
|  | (.0087) | (.0161) | (.0146) | (.0111) | (.0108) | (.0124) |
| Midwest | -.0717*** | -.2359*** | -.1306*** | -.0543*** | -.0041 | .0361*** |
|  | (.0080) | (.0160) | (.0146) | (.0111) | (.0107) | (.0124) |
| Southeast | -.3586*** | -.6387*** | -.4955*** | -.3208*** | -.2268*** | -.1754*** |
|  | (.0061) | (.0106) | (.0096) | (.0073) | (.0071) | (.0081) |
| South | -.3374*** | -.6592*** | -.5269*** | -.3083*** | -.1740*** | -.1108*** |
|  | (.0087) | (.0134) | (.0122) | (.0093) | (.0090) | (.0104) |
| Small municipality | -.1846*** | -.1373*** | -.2139*** | -.1680*** | -.1778*** | -.1686*** |
|  | (.0213) | (.0326) | (.0297) | (.0226) | (.0218) | (.0252) |
| Median municipality | -.1051*** | -.0607*** | -.0737*** | -.1001*** | -.1136*** | -.1131*** |
|  | (.0129) | (.0204) | (.0186) | (.0141) | (.0136) | (.0157) |
| Constant | 1.074*** | 0.741*** | 0.927*** | 1.003*** | 1.178*** | 1.349*** |
|  | (.0260) | (.0414) | (.0377) | (.0286) | (.0277) | (.0319) |
| Number of observations (teams) | 26,954 | 26,954 | 26,954 | 26,954 | 26,954 | 26,954 |

Notes: Values are coefficients (Standard Error). NASF: Family Health Support Centre. hPhysician: working-hour by physicians. hNurse: working-hour by nurses. hDentist: working-hour by dentists. hNurse assistant: working-hour by nurse assistants. hDentist assistant: working-hour by dentist assistants. hCommunity Health Agents: working-hour by community health agents.
