# Supplementary material for: Delivering maternal and childcare at primary healthcare level: The role of PMAQ as a pay for performance strategy in Brazil
Source: PLoS One. 2020 Oct 15;15(10):e0240631. doi: 10.1371/journal.pone.0240631 (PMC7561084; doi:10.1371/journal.pone.0240631)
Supplement: S3 Table — (DOCX) [file pone.0240631.s003.docx]

Table S3. Results from OLS and QR models for average number of consultations for childcare under 2 years old in the 1^st^ Cycle of PMAQ, Brazil

| Variable | PMAQ Cycle 1 | | | | | |
| --- | --- | --- | --- | --- | --- | --- |
|  | OLS | 10^th^ | 25^th^ | 50^th^ | 75^th^ | 90^th^ |
| PMAQ participating | .0033 | .0169*** | .0207*** | .0107** | -.0065 | -.0285** |
|  | (.0039) | (.0028) | (.0033) | (.0046) | (.0069) | (.0112) |
| With dental care | .0085 | .0107** | .0067 | .0094 | .0128 | -.0020 |
|  | (.0069) | (.0052) | (.0061) | (.0085) | (.0127) | (.0207) |
| With NASF | -.0208*** | .0082*** | .0023 | -.0128*** | -.0374*** | -.0585*** |
|  | (.0041) | (.0029) | (.0034) | (.0048) | (.0072) | (.0117) |
| Characteristic of the team |  |  |  |  |  |  |
| hPhysician | .0025*** | -.0004* | .0003 | .0023*** | .0056*** | .0072*** |
|  | (.0003) | (.0002) | (.0003) | (.0004) | (.0005) | (.0009) |
| hNurse | -.0006 | -.0003 | -.0005 | -.0005 | -.0004 | -.0005 |
|  | (.0004) | (.0003) | (.0004) | (.0005) | (.0008) | (.0012) |
| hDentist | .0008*** | .00002 | .0004** | .0006** | .0011** | .0017** |
|  | (.0002) | (.0002) | (.0002) | (.0003) | (.0005) | (.0007) |
| hNurse assistant | .0003*** | .0003*** | .0004*** | .0003*** | .0002* | .0002 |
|  | (.0001) | (.0001) | (.0001) | (.0001) | (.0001) | (.0002) |
| hDentist assistant | -.0007*** | -.0001 | -.0004*** | -.0005*** | -.0009*** | -.0013*** |
|  | (.0002) | (.0001) | (.0002) | (.0002) | (.0003) | (.0005) |
| hCommunity Health Agents | -.0005*** | -.0001*** | -.0003*** | -.0005*** | -.0008*** | -.0009*** |
|  | (.0000) | (.0000) | (.0000) | (.0000) | (.0000) | (.0001) |
| Socioeconomic status |  |  |  |  |  |  |
| Group 1 | .1243*** | .0291** | .0475*** | .0899*** | .1911*** | .3087*** |
|  | (.0163) | (.0123) | (.0143) | (.0200) | (.0300) | (.0489) |
| Group 2 | .1084*** | .0258*** | .0339*** | .0664*** | .1619*** | .2569*** |
|  | (.0108) | (.0088) | (.0103) | (.0144) | (.0215) | (.0351) |
| Group 3 | .1006*** | .0187** | .0325*** | .0542*** | .1514*** | .2340*** |
|  | (.0106) | (.0088) | (.0103) | (.0143) | (.0214) | (.0350) |
| Group 4 | .0699*** | .0233*** | .0259*** | .0305** | .1043*** | .1605*** |
|  | (.0096) | (.0082) | (.0096) | (.0134) | (.0200) | (.0327) |
| Group 5 | .0378*** | .0215*** | .0227*** | .0224*** | .0537*** | .0801*** |
|  | (.0060) | (.0050) | (.0058) | (.0081) | (.0122) | (.0199) |
| Geographic status |  |  |  |  |  |  |
| Rural area | .0467*** | .0062* | .0156*** | .0453*** | .0791*** | .0967*** |
|  | (.0049) | (.0033) | (.0039) | (.0054) | (.0081) | (.0131) |
| North | .0388*** | -.0598*** | -.0466*** | .0381*** | .1147*** | .1427*** |
|  | (.0093) | (.0056) | (.0066) | (.0092) | (.0138) | (.0225) |
| Midwest | -.0698*** | -.0725*** | -.0924*** | -.0772*** | -.0451*** | -.0281 |
|  | (.0082) | (.0056) | (.0066) | (.0092) | (.0137) | (.0224) |
| Southeast | -.0946*** | -.0605*** | -.0739*** | -.0957*** | -.1266*** | -.1334*** |
|  | (.0050) | (.0037) | (.0044) | (.0061) | (.0091) | (.0149) |
| South | -.1399*** | -.0846*** | -.1231*** | -.1456*** | -.1899*** | -.1887*** |
|  | (.0065) | (.0047) | (.0055) | (.0077) | (.0116) | (.0189) |
| Small municipality | -.1124*** | -.0673*** | -.1012*** | -.1305*** | -.1376*** | -.1254*** |
|  | (.0153) | (.0115) | (.0134) | (.0187) | (.0281) | (.0458) |
| Median municipality | -.0686*** | -.0470*** | -.0584*** | -.0612*** | -.0777*** | -.0834*** |
|  | (.0083) | (.0072) | (.0084) | (.0117) | (.0176) | (.0287) |
| Constant | .4782*** | .1605*** | .2912*** | .4384*** | .5456*** | .7577*** |
|  | (.0219) | (.0147) | (.0171) | (.0239) | (.0358) | (.0584) |
| Number of observations (teams) | 26,761 | 26,761 | 26,761 | 26,761 | 26,761 | 26,761 |

Notes: Values are coefficients (Standard Error). NASF: Family Health Support Centre. hPhysician: working-hour by physicians. hNurse: working-hour by nurses. hDentist: working-hour by dentists. hNurse assistant: working-hour by nurse assistants. hDentist assistant: working-hour by dentist assistants. hCommunity Health Agents: working-hour by community health agents.
