# Supplementary material for: Delivering maternal and childcare at primary healthcare level: The role of PMAQ as a pay for performance strategy in Brazil
Source: PLoS One. 2020 Oct 15;15(10):e0240631. doi: 10.1371/journal.pone.0240631 (PMC7561084; doi:10.1371/journal.pone.0240631)
Supplement: S4 Table — (DOCX) [file pone.0240631.s004.docx]

Table S4. Results from OLS and QR models for the number of physician consultations for children under 1 year old in the 1^st^ Cycle of PMAQ, Brazil

| Variable | PMAQ Cycle 1 | | | | | |
| --- | --- | --- | --- | --- | --- | --- |
|  | OLS | 10^th^ | 25^th^ | 50^th^ | 75^th^ | 90^th^ |
| PMAQ participating | .0113*** | .0091*** | .0130*** | .0126*** | .0145*** | -.0038 |
|  | (.0028) | (.0016) | (.0021) | (.0027) | (.0045) | (.0083) |
| With dental care | .0003 | .0037 | .0025 | .0024 | .0078 | -.0170 |
|  | (.0054) | (.0029) | (.0038) | (.0049) | (.0083) | (.0153) |
| With NASF | -.0080*** | .0031* | .0036 | -.0011 | -.0139*** | -.0270*** |
|  | (.0029) | (.0017) | (.0022) | (.0028) | (.0047) | (.0087) |
| Characteristic of the team |  |  |  |  |  |  |
| hPhysician | .0040*** | .0006*** | .0018*** | .0041*** | .0069*** | .0096*** |
|  | (.0003) | (.0001) | (.0002) | (.0002) | (.0004) | (.0006) |
| hNurse | .0008** | -.0002 | .0002 | .0005* | .0016*** | .0026*** |
|  | (.0004) | (.0002) | (.0002) | (.0003) | (.0005) | (.0009) |
| hDentist | .0006*** | .00003 | -.00003 | .0004** | .0008*** | .0008 |
|  | (.0002) | (.0001) | (.0001) | (.0002) | (.0003) | (.0005) |
| hNurse assistant | .0006*** | .0003*** | .0005*** | .0007*** | .0007*** | .0006*** |
|  | (.0001) | (.0000) | (.0000) | (.0001) | (.0001) | (.0002) |
| hDentist assistant | -.0003** | .00002 | .0001 | -.0003** | -.0006*** | -.0005 |
|  | (.0001) | (.0001) | (.0001) | (.0001) | (.0002) | (.0004) |
| hCommunity Health Agents | -.0005*** | -.0001*** | -.0002*** | -.0004*** | -.0005*** | -.0008*** |
|  | (.00002) | (.00001) | (.00001) | (.00002) | (.00003) | (.00005) |
| Socioeconomic status |  |  |  |  |  |  |
| Group 1 | -.0094 | .0279*** | .0391*** | -.0022 | -.02095 | -.1185*** |
|  | (.0150) | (.0069) | (.0091) | (.0116) | (.0198) | (.0363) |
| Group 2 | -.0388*** | .0119** | .0154** | -.0219*** | -.0516*** | -.1418*** |
|  | (.0099) | (.0050) | (.0065) | (.0083) | (.0142) | (.0260) |
| Group 3 | -.0592*** | .0027 | .0004 | -.0431*** | -.0749*** | -.1841*** |
|  | (.0099) | (.0050) | (.0065) | (.0083) | (.0141) | (.0259) |
| Group 4 | -.0737*** | -.0073 | -.0155** | -.0597*** | -.0889*** | -.1812*** |
|  | (.0096) | (.0046) | (.0061) | (.0077) | (.0132) | (.0241) |
| Group 5 | -.0521*** | -.00003 | -.0086** | -.0371*** | -.0683*** | -.1415*** |
|  | (.0056) | (.0028) | (.0037) | (.0047) | (.0080) | (.0146) |
| Geographic status |  |  |  |  |  |  |
| Rural area | .0231*** | .0143*** | .0216*** | .0263*** | .0362*** | .0371*** |
|  | (.0031) | (.0019) | (.0025) | (.0031) | (.0053) | (.0097) |
| North | .0533*** | .0007 | .0133*** | .0416*** | .0750*** | .1291*** |
|  | (.0053) | (.0032) | (.0042) | (.0053) | (.0091) | (.0166) |
| Midwest | .1344*** | .0442*** | .0796*** | .1178*** | .1726*** | .2218*** |
|  | (.0060) | (.0032) | (.0042) | (.0053) | (.0091) | (.0167) |
| Southeast | .0692*** | -.0114*** | .0094*** | .0537*** | .1108*** | .1656*** |
|  | (.0037) | (.0021) | (.0028) | (.0035) | (.0060) | (.0110) |
| South | .0978*** | -.0047* | .0256*** | .0739*** | .1551*** | .2334*** |
|  | (.0054) | (.0027) | (.0035) | (.0045) | (.0076) | (.0140) |
| Small municipality | .0457*** | .0148** | .0086 | .0412*** | .0652*** | .1369*** |
|  | (.0142) | (.0065) | (.0086) | (.0109) | (.0185) | (.0340) |
| Median municipality | .01108 | .0120*** | .0005 | .0095 | .0101 | .0477** |
|  | (.0085) | (.0041) | (.0053) | (.0068) | (.0116) | (.0212) |
| Constant | .1362*** | .0101 | .0181* | .0481*** | .0680*** | .2290*** |
|  | (.0213) | (.0083) | (.0108) | (.0138) | (.0235) | (.0431) |
| Number of observations (teams) | 27,070 | 27,070 | 27,070 | 27,070 | 27,070 | 27,070 |

Notes: Values are coefficients (Standard Error). NASF: Family Health Support Centre. hPhysician: working-hour by physicians. hNurse: working-hour by nurses. hDentist: working-hour by dentists. hNurse assistant: working-hour by nurse assistants. hDentist assistant: working-hour by dentist assistants. hCommunity Health Agents: working-hour by community health agents.
