# Supplementary material for: Delivering maternal and childcare at primary healthcare level: The role of PMAQ as a pay for performance strategy in Brazil
Source: PLoS One. 2020 Oct 15;15(10):e0240631. doi: 10.1371/journal.pone.0240631 (PMC7561084; doi:10.1371/journal.pone.0240631)
Supplement: S5 Table — (DOCX) [file pone.0240631.s005.docx]

Table S5. Results from the OLS and QR models for antenatal consultations in the 1^st^ Cycle of PMAQ (missing values imputed), Brazil

| Variable | PMAQ Cycle 1 | | | | | |
| --- | --- | --- | --- | --- | --- | --- |
|  | OLS | 10^th^ | 25^th^ | 50^th^ | 75^th^ | 90^th^ |
| PMAQ participating | .0546*** | .0888*** | .0823*** | .0521*** | .0267*** | .0096 |
|  | (.0042) | (.0079) | (.0068) | (.0052) | (.0049) | (.0059) |
| Additional controls | Yes | Yes | Yes | Yes | Yes | Yes |
| Number of observations (teams) | 33,368 | 33,368 | 33,368 | 33,368 | 33,368 | 33,368 |
| Note: Values are coefficients (Standard Error). | | |  |  |  |  |
