# Supplementary material for: Delivering maternal and childcare at primary healthcare level: The role of PMAQ as a pay for performance strategy in Brazil
Source: PLoS One. 2020 Oct 15;15(10):e0240631. doi: 10.1371/journal.pone.0240631 (PMC7561084; doi:10.1371/journal.pone.0240631)
Supplement: S7 Table — (DOCX) [file pone.0240631.s007.docx]

Table S7. Results from OLS and QR models for the number of physician consultations for children under 1 year old in the 1^st^ Cycle of PMAQ (missing values imputed), Brazil

| Variable |  | PMAQ Cycle 1 | | | | |
| --- | --- | --- | --- | --- | --- | --- |
|  | OLS | 10^th^ | 25^th^ | 50^th^ | 75^th^ | 90^th^ |
| PMAQ participating | .0121*** | .0108*** | .0158*** | .0136*** | .0132*** | -.0069 |
|  | (.0027) | (.0017) | (.0022) | (.0026) | (.0044) | (.0074) |
| Additional controls | Yes | Yes | Yes | Yes | Yes | Yes |
| Number of observations (teams) | 33,368 | 33,368 | 33,368 | 33,368 | 33,368 | 33,368 |
| Note: Values are coefficients (Standard Error). | | |  |  |  |  |
