# Supplementary material for: Delivering maternal and childcare at primary healthcare level: The role of PMAQ as a pay for performance strategy in Brazil
Source: PLoS One. 2020 Oct 15;15(10):e0240631. doi: 10.1371/journal.pone.0240631 (PMC7561084; doi:10.1371/journal.pone.0240631)
Supplement: S8 Table — (DOCX) [file pone.0240631.s008.docx]

Table S8. Results from the OLS and QR models for antenatal consultations in the 2^nd^ Cycle of PMAQ (missing values imputed), Brazil

| Variable |  | PMAQ Cycle 2 | | | | |
| --- | --- | --- | --- | --- | --- | --- |
|  | OLS | 10^th^ | 25^th^ | 50^th^ | 75^th^ | 90^th^ |
| PMAQ participating | .0982*** | .1314*** | .1365*** | .1025*** | .0694*** | .0314*** |
|  | (.0055) | (.0113) | (.0083) | (.0066) | (.0071) | (.0085) |
| Additional controls | Yes | Yes | Yes | Yes | Yes | Yes |
| Number of observations (teams) | 39,211 | 39,211 | 39,211 | 39,211 | 39,211 | 39,211 |
| Note: Values are coefficients (Standard Error). | | |  |  |  |  |
