# Supplementary material for: Delivering maternal and childcare at primary healthcare level: The role of PMAQ as a pay for performance strategy in Brazil
Source: PLoS One. 2020 Oct 15;15(10):e0240631. doi: 10.1371/journal.pone.0240631 (PMC7561084; doi:10.1371/journal.pone.0240631)
Supplement: Attachment — Submitted filename: response_to_reviewers.docx [file pone.0240631.s011.docx]

Dear Editor,

We thank you for the opportunity to have our manuscript **PONE-D-20-14125**, entitled **"Delivering maternal and childcare at primary healthcare level: the role of PMAQ as a pay for performance strategy in Brazil"**, evaluated for publication by Plos One. After analyzing all the comments made, we are happy to implement the suggestions to improve the manuscript, as described below.

Yours sincerely,

The Authors

Table 1: Summary

| Questions | **Reviewer 1** | **Reviewer 2** | **Reviewer 3** | **Reviewer 4** |
| --- | --- | --- | --- | --- |
| Is the manuscript technically sound, and do the data support the conclusions? | Yes | Yes | Partly | Partly |
| Has the statistical analysis been performed appropriately and rigorously? | Yes | Yes | I Don't Know | I Don't Know |
| Have the authors made all data underlying the findings in their manuscript fully available? | Yes | Yes | Yes | Yes |
| Is the manuscript presented in an intelligible fashion and written in standard English? | Yes | Yes | No | Yes |
| Review Comments to the Author | See below | See below | See below | See below |
| Do you want your identity to be public for this peer review? | No | No | Blami Dao | No |

**Reviewer 1, comment 1:** “Over the last decade, there has been an increase in the use of pay for performance (P4P) strategies as an approach for improving availability and utilization of healthcare services. In this article, authors Medeiros et al. explore the impact of (P4P) at a national level throughout Brazil on the provision of maternal and child health care. Specifically, the authors use a cross sectional quantile regression analysis to assess the association between P4P, or the strategy of incentivizing providers to delivery key services, and the provision of key maternal and child health services across national Family Health Teams. Authors explored the association over time, analyzing 33,368 Family Health Teams between 2011-2012 (Cycle 1) and 39,211 teams between 2013-2014 (Cycle 2). Key findings from the study include: a positive association between P4P participation and antenatal visits per month, with an increased effect in lower quantiles; a positive association between P4P participation and the number of visits for children under two per month in the lower quantiles; and a positive association between P4P and visits for children under one in the lower tail. Results also indicated that there were considerable portions of the teams that did not achieve national recommendations for maternal and child health visits. The current manuscript requires some additional edits and considerations for added context; please see below for specific suggestions.

**Our Response:** We would like to thank you for your consideration and time spent reviewing our manuscript.

**Reviewer 1, comment 2:** “This comes up a bit in the discussion, through the comparison of findings to existing literature, but it may strengthen the argument for the need for this study to provide – upfront in the introduction – a discussion of any ethical considerations associated with a P4P system. For example, do increased visits under P4P lead to increased quality of care or improved outcomes? The data used in the study does not allow the authors to identify an association between P4P and clinical outcomes, but consider including a more comprehensive review of the P4P literature in the introduction, rather than the majority of this literature residing in the discussion section.”

**Our response:** As suggested by the reviewer, we added ethical considerations associated with P4P, including equity concerns.

“There are also ethical issues and concerns regarding its impact on deprived population groups leading to worsening health inequalities. P4P programmes oftentimes focus only on process and structural indicators without measuring the effect on outcomes, such as mortality (8).”

**Reviewer 1, comment 3:** “It may be useful to the reader for the authors to provide a more detailed discussion of how covariates for the model were selected. The manuscript provides an appropriate description of the indicators included in the model, but providing additional detail and context on selection would be helpful. For example, were the covariates selected based on theory? Previous research? Existing literature?”

**Our response:** Our model is based on both theorical and empirical literature. We used three set of covariates, including socioeconomic, geographic and team characteristics. Socioeconomic and geographic characteristics are well-documented in the literature since they play some role on health outcomes (1, 2). We included team characteristics because the composition of teams can vary across the country (3). All teams need to have at least a physician, a nurse, a nurse assistant, and a community health agent. That is the minimum composition, but municipalities can hire more health workers depending on their budget and availability of the local health worker supply (because that, we controlled for working-hours by health worker). Teams can also include other health workers (not mandatory), such as dentists and dentist assistant (Dental Care) or any other health workers besides the ones listed previously (NASF) (4).

1. Viegas Andrade M, Noronha K, Singh A, Rodrigues CG, Padmadas SS. Antenatal care use in Brazil and India: scale, outreach and socioeconomic inequality. Health & place. 2012;18(5):942-50.
2. Lansky S, Lima Friche AA, Silva AA, Campos D, Azevedo Bittencourt SD, Carvalho ML, et al. Birth in Brazil survey: neonatal mortality, pregnancy and childbirth quality of care. Cadernos de saude publica. 2014;30 Suppl 1:S1-15.
3. BRASIL. Política Nacional de Atenção Básica. Brasília: Ministério da Saúde; 2012.
4. Abrasco. Contribution to a strategic policy agenda for Primary Health Care in the Brazilian Unified Health System (SUS). Saúde Debate. 2018;42(1).

**Reviewer 1, comment 4: “**Additional, more minor areas where it would be helpful to address edits include: (1) For clarity purposes, it may be helpful to add an additional sentence or two describing the structure of the Family Health Teams. For readers unfamiliar with the Brazil health system, the relationship between Family Health Strategy and Primary Health Teams within Family Health Teams is likely unknown.”

**Our response:** As suggested by the reviewer, we include a new sentence.

“FHTs provide coordinated, comprehensive, and continuous care through multidisciplinary teams composed of physicians, nurses, nurse assistants and community health agents.

**Reviewer 1, comment 5:** “(2) Acronyms are not consistent throughout the manuscript. There are instances where acronyms are previously defined, and then the full term is used later on throughout the paper. This was most commonly seen for pay for performance (P4P), Ministry of Health (MOH), and Family Health Strategy (FHS).”

**Our response:** As suggested by the reviewer, we corrected the inconsistences related to acronyms.

**Reviewer 1, comment 6:** “(3) At the end of the introduction, the authors describe the key outcomes being measured in the analysis, indicating that they were selected for clinical purposes, as they are essential for good practice in MCH. Consider providing a few additional sentences more clearly linking the selected outcomes with clinical outcomes, based on what is known in the literature, to clarify to the reader why these were selected.”

**Our response:** As suggested by the reviewer, we included new sentences to clarify the link between the selected outcomes and clinical outcomes.

“For example, in sub-Saharan African countries, there is a 39% lower risk of neonatal mortality among women who attended at least one antenatal care visit during pregnancy (24). In Brazil, 40% of the avoidable neonatal deaths can be attributed to poor antenatal care (25). Additionally, three consultations in the first year of life reduces the risk of neonatal mortality by 20.5% in Brazil (26).”

**Reviewer 1, comment 7:** “(4) Within the Participants section of the manuscript, the authors describe the fact that there was significant data missing from the dataset used for both cycles. However, this does not seem to be mentioned in the discussion as a limitation. Consider discussing the potential impact of this missing data as a limitation.”

**Our response:** We employed a method of multiple imputation and re-estimated the OLS and QR models. We included these estimated in the Supplementary Material. We include this procedure in the method and new sentences in the results and discussion.

“Missing data is a concern especially for inflexible multivariable methods (31). In addition to the main analysis, we employed the method for multiple imputation and re-estimated the models as a form of sensitivity analysis. This is a well-known approach for dealing with the loss of observations, with the missing values imputed based on an iterative method.” (Methods)

“Tables S5-S10 present our results considering the imputation for missing values. The results were similar to those estimated in the main analysis.” (Results)

“However, similar results were obtained when we employed a method for multiple imputation.” (Discussion)

| Variable |  | Observations | | | | | | |
| --- | --- | --- | --- | --- | --- | --- | --- | --- |
|  |  | Cycle 1 | | |  | Cycle 2 | | |
|  |  | Complete | Imputed | Total |  | Complete | Imputed | Total |
| cAntenatal |  | 30,819 | 2,549 | 33,368 |  | 35,410 | 3,801 | 39,211 |
| cChildcare under 2 years old | | 30,556 | 2,812 | 33,368 |  | 34,705 | 4,506 | 39,211 |
| cPhysician for children under 1 year | | 30,964 | 2,404 | 33,368 |  | 35,636 | 3,575 | 39,211 |
| hPhysician |  | 29,628 | 3,740 | 33,368 |  | 37,190 | 2,021 | 39,211 |
| hNurse |  | 29,628 | 3,740 | 33,368 |  | 37,190 | 2,021 | 39,211 |
| hDentist |  | 29,628 | 3,740 | 33,368 |  | 37,190 | 2,021 | 39,211 |
| hNurse assistant |  | 29,628 | 3,740 | 33,368 |  | 37,190 | 2,021 | 39,211 |
| hDentist assistant |  | 29,628 | 3,740 | 33,368 |  | 37,190 | 2,021 | 39,211 |
| hCommunity Health Agents | | 29,628 | 3,740 | 33,368 |  | 37,190 | 2,021 | 39,211 |
| Rural area |  | 32,661 | 707 | 33,368 |  | 38,388 | 823 | 39,211 |
| With dental care |  | 33,360 | 8 | 33,368 |  | 39,202 | 9 | 39,211 |

Table S5. Results from the OLS and QR models for antenatal consultations in the 1st Cycle of PMAQ (missing values imputed), Brazil

| Variable | PMAQ Cycle 1 | | | | | |
| --- | --- | --- | --- | --- | --- | --- |
|  | OLS | 10th | 25th | 50th | 75th | 90th |
| PMAQ participating | .0546*** | .0888*** | .0823*** | .0521*** | .0267*** | .0096 |
|  | (.0042) | (.0079) | (.0068) | (.0052) | (.0049) | (.0059) |
| Additional controls | Yes | Yes | Yes | Yes | Yes | Yes |
| Number of observations (teams) | 33,368 | 33,368 | 33,368 | 33,368 | 33,368 | 33,368 |
| Note: Values are coefficients (Standard Error). | | |  |  |  |  |

Table S6. Results from OLS and QR models for average number of consultations for childcare under 2 years old in the 1st Cycle of PMAQ (missing values imputed), Brazil

| Variable | PMAQ Cycle 1 | | | | | |
| --- | --- | --- | --- | --- | --- | --- |
|  | OLS | 10th | 25th | 50th | 75th | 90th |
| PMAQ participating | .0037 | .0179*** | .0206*** | .0103** | -.0063 | -.0264*** |
|  | (.0037) | (.0027) | (.0033) | (.0044) | (.0065) | (.0100) |
| Additional controls | Yes | Yes | Yes | Yes | Yes | Yes |
| Number of observations (teams) | 33,368 | 33,368 | 33,368 | 33,368 | 33,368 | 33,368 |
| Note: Values are coefficients (Standard Error). | | |  |  |  |  |

Table S7. Results from OLS and QR models for the number of physician consultations for children under 1 year old in the 1st Cycle of PMAQ (missing values imputed), Brazil

| Variable |  | PMAQ Cycle 1 | | | | |
| --- | --- | --- | --- | --- | --- | --- |
|  | OLS | 10th | 25th | 50th | 75th | 90th |
| PMAQ participating | .0121*** | .0108*** | .0158*** | .0136*** | .0132*** | -.0069 |
|  | (.0027) | (.0017) | (.0022) | (.0026) | (.0044) | (.0074) |
| Additional controls | Yes | Yes | Yes | Yes | Yes | Yes |
| Number of observations (teams) | 33,368 | 33,368 | 33,368 | 33,368 | 33,368 | 33,368 |
| Note: Values are coefficients (Standard Error). | | |  |  |  |  |

Table S8. Results from the OLS and QR models for antenatal consultations in the 2nd Cycle of PMAQ (missing values imputed), Brazil

| Variable |  | PMAQ Cycle 2 | | | | |
| --- | --- | --- | --- | --- | --- | --- |
|  | OLS | 10th | 25th | 50th | 75th | 90th |
| PMAQ participating | .0982*** | .1314*** | .1365*** | .1025*** | .0694*** | .0314*** |
|  | (.0055) | (.0113) | (.0083) | (.0066) | (.0071) | (.0085) |
| Additional controls | Yes | Yes | Yes | Yes | Yes | Yes |
| Number of observations (teams) | 39,211 | 39,211 | 39,211 | 39,211 | 39,211 | 39,211 |
| Note: Values are coefficients (Standard Error). | | |  |  |  |  |

Table S9. Results from OLS and QR models for average number of consultations for childcare under 2 years old in the 2nd Cycle of PMAQ (missing values imputed), Brazil

| Variable | PMAQ Cycle 2 | | | | | |
| --- | --- | --- | --- | --- | --- | --- |
|  | OLS | 10th | 25th | 50th | 75th | 90th |
| PMAQ participating | -.0028 | .0221*** | .0270*** | .0121** | -.0265*** | -.0460*** |
|  | (.0049) | (.0037) | (.0044) | (.0054) | (.0085) | (.0125) |
| Additional controls | Yes | Yes | Yes | Yes | Yes | Yes |
| Number of observations (teams) | 39,211 | 39,211 | 39,211 | 39,211 | 39,211 | 39,211 |
| Note: Values are coefficients (Standard Error). | | |  |  |  |  |

Table S10. Results from OLS and QR models for the number of physician consultations for children under 1 year old in the 2nd Cycle of PMAQ (missing values imputed), Brazil

| Variable | PMAQ Cycle 2 | | | | | |
| --- | --- | --- | --- | --- | --- | --- |
|  | OLS | 10th | 25th | 50th | 75th | 90th |
| PMAQ participating | .00987*** | .01191*** | .01543*** | .01485*** | .00036 | -.01471 |
|  | (.0037) | (.0025) | (.0028) | (.0037) | (.0060) | (.0115) |
| Additional controls | Yes | Yes | Yes | Yes | Yes | Yes |
| Number of observations (teams) | 39,211 | 39,211 | 39,211 | 39,211 | 39,211 | 39,211 |
| Note: Values are coefficients (Standard Error). | | |  |  |  |  |

**Reviewer 1, comment 8:** “(5) Finally, this manuscript would benefit from an overall copy edit to address any minor spelling or grammatical errors.”

**Our response:** The manuscript was proofread by an English native speaker.

**Reviewer 2, comment 1:** “This is an interesting study, with the limitations that the Authors well acknowledged in Discussion. I have no idea of neonatal death numbers in Brazil, and whether these number would change as a result of P4P, at least in the most disadvantaged areas.”

**Our Response:** We would like to thank you for your consideration and time spent reviewing our manuscript. You have raised an important question (the association of neonatal deaths with P4P). Unfortunately, we can not answer it because mortality data is not available at team level. The least disaggregated level provided by the Ministry of Health is the municipality.

**Reviewer 3, comment 1:** “This paper addresses an important topic which the impact of the pay for performance policy on access to quality MCH services.
My main concern is about the statistical methods used to analyze the data. I am not familiar with those methods and I suggest that PLoS One hires a statistician to review that part of the paper.”

**Our Response:** We would like to thank you for your consideration and time spent reviewing our manuscript.

**Reviewer 4, comment 1:** “This manuscript represents a comparative analysis of maternal and child service utilization of a pay for performance program for primary care providers in Brazil from 2001 - 2004. Overall, this is a well written manuscript and the information is timely given the expansion of pay for performance programs. However, there are some gaps in the methods that should be mentioned earlier or adjusted in the analysis, specifically overlap in outcome measures for child care visits at both one and two years and the potential omission of immunization visits. The manuscript would also benefit from review by a native English speaker for a few areas with subject-verb agreement issues or incorrect work choice or syntax.”

**Our Response:** We would like to thank you for your consideration and time spent reviewing our manuscript.

**Reviewer 4, comment 2:** “Introduction: The Introduction is well-reasoned and provides insight into the context. Of note, the Introduction indicates that the PMAQ third cycle still had not achieved complete coverage”.

**Our response:** Although PMAQ has expanding fairly rapid over the three cycles, it did not reach 100% coverage. To the best of our knowledge, there is no survey that sought to identify the reasons why some teams opted to not participate in PMAQ. Some teams may be averse to evaluation processes. Or the municipality chose to opt out, which has the final decision about participating in PMAQ.

**Reviewer 4, comment 3:** “Methods: The description of the analysis is fairly complete but it is unclear why the authors selected the dates ranges for time as remote as 2014 – it is now six years later and it would be helpful to see longer-term trends, assuming this program continued. Please add detail for why these time bands were selected and why the analysis was limited to only two cycles.”

**Our response:** PMAQ underwent several changes over the three cycles, particularly in terms of maternal and childcare. The outcomes used in our model (consultations for antenatal, children under 1 and children under 2) were withdrawn from the third cycle. They are no more incentivized. There is only one indicator related to maternal and childcare in the third cycle: “percentage of new-borns that received care in the first week of life”. Moreover, the information system used by the Ministry of Health to verify the production of each team also changed (SIAB in the first two cycles and e-SUS in the third one). There is no comparable data between first/second cycles and third one related to maternal and childcare. Based on that, we decided to not include the third cycle in our study.

**Reviewer 4, comment 4:** “Methods: The outcomes of child care visits for children under one year and a separate variable for children under two years reflect overlap and would likely be collinear if used in the same models.”

**Our response:** In fact, some overlapping can emerge between the two variables. Based on that, we used them in separate models to avoid collinearity as mentioned by the reviewer. We decided to include both variables because consultation under 1 year are performed exclusively by physicians and consultations under 2 year are performed by physicians and nurses. Know the results from these two variables may be of interesting of decision makers.

**Reviewer 4, comment 5:** “Methods: This is a minor point but I would be careful regarding the use of the word “dummy” for variables. It is reasonable to categorize variables for expediency as long as the categories are defined and they do not need to be referred to as “dummy variables” unless they represent multiple measures within one variable, which is not the case here.”

**Our response:** The use of “dummy” to indicate a variable that assumes zero or one values seems to be a standard practice in economics and public health literature, as you can see in some papers published in Plos One and other journals. We would like to keep the word dummy.

1. Wolf RT, Jeppesen P, Gyrd-Hansen D, Group CCCS, Oxholm AS. Evaluation of a screening algorithm using the Strengths and Difficulties Questionnaire to identify children with mental health problems: A five-year register-based follow-up on school performance and healthcare use. PloS one. 2019;14(10):e0223314.
2. Jolly SP, Chowdhury TR, Rahman M, Alam A, Afsana K. Prevention of social exclusion and role of antenatal care by BRAC community health workers in improving safe motherhood and neonatal care in urban slums of Bangladesh. PloS one. 2020.
3. Chan CL, Lin W, Yang NP, Lai KR, Huang HT. Pre-emergency-department care-seeking patterns are associated with the severity of presenting condition for emergency department visit and subsequent adverse events: a timeframe episode analysis. PloS one. 2015;10(6):e0127793.
4. Garcia ACP, Lima RdCD, Galavote HS, Coelho APS, Vieira ECL, Silva RC, et al. Community Health Agent: from the profile to activities done. Trabalho, Educação e Saúde. 2016;15(1).

**Reviewer 4, comment 6:** Results: Figure 1 would benefit from clear labeling of all content – please remember the adage that figures and tables should be able to stand on their own with needing text to describe the findings.”

**Our response:** As suggested by the reviewer, we reworded the labelling of Fig 1. We also added a note to explaining the figure.

“Figure 1. Cumulative distribution of average consultations per month for maternal and childcare performed by PMAQ participating and non-participating teams in cycles 1 and 2, Brazil, 2011-2014

Note: Number of consultations per month recommended by the Ministry of Health (red line). Central line shows median achievement and box shows interquartile range.”

**Reviewer 4, comment 7:** “Results: In the tables, please limit results to only 1 decimal point in Table 1 and no more than three decimal places in Tables 2-4. The sheer volume of numbers make the data very difficult to interpret.”

**Our response:** In Table 1, we kept three decimal places for numbers less than 1, and one decimal place for numbers greater than 1. In Tables 2-4, we used 4 decimal places for numbers less than 1, since some coefficients are very low.

**Reviewer 4, comment 8:** “Results: I question whether including dental services and providers or the mental health teams is worthwhile in this analysis – how do the authors see this impacting MCH service utilization? More parsimonious models would be preferable.”

**Our response:** Although the Family Health Team is the main strategy to delivery primary health care in Brazil, it could be complemented by other teams, such as oral care and Family Health Support Unit (NASF). When all these teams work together in a multidisciplinary way, a more comprehensive care can be delivered to pregnant women and infants. We bring some data that might corroborate our decision to include oral care and NASF in our model. The prevalence of postpartum depression among healthy mothers without a prior history of depression is 17% (1). NASF can also hire nutritionists, who can integrate diet and nutrition into paediatric primary care, preventing weight gain and obesity (2). Oral health is part of overall health. Pregnant women are more likely to have oral problems due to various hormonal changes and fluctuations in intraoral flora; around 40% of pregnant women suffer from some form of periodontitis and up to 10% may develop pregnancy oral tumours (3).

1. Shorey S, Chee CYI, Ng ED, Chan YH, Tam WWS, Chong YS. Prevalence and incidence of postpartum depression among healthy mothers: A systematic review and meta-analysis. J Psychiatr Res. 2018;104:235-248. doi:10.1016/j.jpsychires.2018.08.001
2. Harrison SE, Greenhouse D. Dietary and Nutrition Recommendations in Pediatric Primary Care: A Call to Action. South Med J. 2018;111(1):12-17. doi:10.14423/SMJ.0000000000000754
3. Abou El Fadl, R., Blair, M., & Hassounah, S. (2016). Integrating Maternal and Children's Oral Health Promotion into Nursing and Midwifery Practice- A Systematic Review. PloS one, 11(11), e0166760. https://doi.org/10.1371/journal.pone.0166760

**Reviewer 4, comment 9:** “Results: The same question asked earlier about overlap between children under 1 and children under 2 is more prominent here and the reader is left wondering what the explanation is for such a difference between these two outcomes with respect to coverage.”

**Our response:** As mentioned previously (Reviewer 4, comment 4), some overlapping may occur related to consultations provided by physicians for the children’s first year of life. However, consultations for children under 2 years also include nurses. The lower coverage of consultations for children under 2 years may be associated with lower engagement of nurses, but this must be confirmed in future studies.

**Reviewer 4, comment 10:** “Discussion: Overall, the Discussion is comprehensive but the Limitations section mentions a major limitation that should be raised in Methods. Immunization visits are often the bulk of well-child visits, particularly in the first year. Having these visits potentially excluded from the analysis may result in substantial bias and should be mentioned in Methods as well.”

**Our response:** As suggested by the reviewer, we included an additional sentence in the methods.

“It is worth noting that childcare includes consultations for routine growth and development monitoring and excludes acute care and immunization programmes.”

**Reviewer 4, comment 11:** “Discussion: The mention that community health agent workforce was associated with fewer visits deserves greater attention as this seems counter-intuitive – was there any care provided at community level that may have resulted in not visiting the facility?”

**Our response:** There are few studies about the services delivered by community health agents at national level in Brazil. A local study, which analysed data from 121 community health agents in ten municipalities, showed that 41.3% and 19.0% of the sample provided care to pregnant women and children, respectively (1). The most frequent activities were home visit (90,1%) and situation analysis of the catchment families (95.0%), both in terms of health problems and vulnerability (1). This may suggest the maternal and childcare are not the most priority groups for community health agents, since they have many other duties.

1. Garcia ACP, Lima RCD, Galavote HS, Coelho APS et al. Agente comunitário de saúde no espírito santo: do perfil às atividades desenvolvidas. Trabalho, Educação e Saúde, 15(1), 283-300. Epub December 08, 2016. <https://doi.org/10.1590/1981-7746-sol00039>
