# Supplementary material for: Delivering maternal and childcare at primary healthcare level: The role of PMAQ as a pay for performance strategy in Brazil
Source: PLoS One. 2020 Oct 15;15(10):e0240631. doi: 10.1371/journal.pone.0240631 (PMC7561084; doi:10.1371/journal.pone.0240631)
Supplement: Attachment — Submitted filename: response_to_reviewers.docx [file pone.0240631.s012.docx]

Dear Editor,

We thank you for the opportunity to have our manuscript **PONE-D-20-14125R1**, entitled **"Delivering maternal and childcare at primary healthcare level: the role of PMAQ as a pay for performance strategy in Brazil"**, evaluated for publication by Plos One.

We would like to thank reviewers for their consideration and time spent reviewing our manuscript.

Yours sincerely,

The Authors

**Reviewer 3, comment 1:** “Can the author do the following changes to the manuscript?

- in the results section, instead of describing the results by Figure 1 shows.., table 1 shows.., he should make a sentence and put the figure or table number into brackets
- at the end, instead of final considerations, he/she should put conclusions”

**Our response:** Thank you for taking the time to review our manuscript and for the helpful comments you have provided. Changes are added to the manuscript as suggested by the reviewer.

1. Text removed in **Results**:

“Table 1 shows the descriptive statistics of the variables used in this study based on the mean and standard deviation.”

2. Text removed in **Results**:

“Figure 1 shows the”

3. Text removed in **Results**:

“Table 2 presents the results from the OLS and QR models for antenatal consultations in the period related to the second cycle of PMAQ. As expected, the”

4. Text removed in **Results**:

“Table 3 presents the results from OLS and QR models for average number of consultations for childcare under 2 years per month in the second cycle of PMAQ. On the one hand.”

5. Text removed in **Results**:

“Table 4 presents the results from OLS and QR models for the number of physician consultations for children under 1 year in the second cycle of PMAQ.”

6. Text removed in **Conclusion**:

“Final Considerations”.

**Reviewer 4, comment 1:** “While I appreciate the time taken by the authors to write responses to the points raised in review, very few of the suggestions to improve the manuscript were taken on board. This lack of responsiveness in the manuscript occurred even when the authors agreed with the point raised, such as excluding immunization visits, which should be mentioned more prominently as a source of bias and potential care at the community level, which should be raised in the Discussion. Reviewers spend quite a bit of time as volunteers to evaluate and recommend ways to improve manuscripts. In my view, the authors need to integrate more of these recommendations, particularly surrounding the analysis, potential biases, presentation of values in the tables, and quality of the writing, before this manuscript can be accepted.”

**Our response:** Thank you for taking the time to review our manuscript and for the helpful comments you have provided. We do recognize the valuable role of peer review in improving the quality of scientific publication. In the previous round of the review, many replies were not include in the manuscript due to misperception that “response to reviewers” is part on the paper, such as supplementary material, since Plos One policy is to publish the peer review history. Sorry for this misperception. At this round, we included all our replies in the manuscript. The following texts were included in the manuscript, based on the previous round of the review:

7. Text included in **Method – Study setting**:

“When all these teams work together in a multidisciplinary way, a more comprehensive care can be delivered to pregnant women and infants. The prevalence of postpartum depression among healthy mothers without a prior history of depression is 17% (31). NASF can also hire nutritionists, who can integrate diet and nutrition into paediatric primary care, preventing weight gain and obesity (32). Oral health is also part of overall health. Pregnant women are more likely to have oral problems due to various hormonal changes and fluctuations in intraoral flora; around 40% of pregnant women suffer from some form of periodontitis and up to 10% may develop pregnancy oral tumours (33).”

8. Text included in **Methods – Study setting**:

“PMAQ underwent several changes over the three cycles, particularly in terms of maternal and childcare. The outcomes used in our model (consultations for antenatal, children under 1 and children under 2) were removed from the third cycle. They are no more incentivized. There is only one indicator related to maternal and childcare in the third cycle: “percentage of new-borns that received care in the first week of life”. Moreover, the information system used by the MoH to verify the production of each team also changed (SIAB in the first two cycles and e-SUS in the third one). There is no comparable data between first/second cycles and third one related to maternal and childcare. Based on that, we decided to not include the third cycle in our study.”

9. Text included in **Methods – Data source and variables**:

“Acute care and immunization were not included in our model because they were not available at FHT level. Additionally, the outcomes of childcare visits for children under 1 year and under 2 may reflect overlap. Based on that, we used them in separate models to avoid collinearity. We decided to include both variables because consultation under 1 year are performed exclusively by physicians and consultations under 2 year are performed by physicians and nurses.”

10. Text included in **Discussion**:

“There are few studies about the services delivered by community health agents at national level in Brazil. A local study, which analysed data from 121 community health agents in ten municipalities, showed that 41.3% and 19.0% of the sample provided care to pregnant women and children, respectively (56). The most frequent activities were home visit (90,1%) and situation analysis of the catchment families (95.0%), both in terms of health problems and vulnerability (56). This may suggest the maternal and childcare are not a high-priority group for community health agents, since they have many other duties.”

11. Text included in **Discussion**:

Fourth, another potential source of bias is that not all maternal and child care were captured in our study e.g. acute care and immunization programmes. Based on that, our results may underestimate the role of PHT in delivering maternal and child care. We investigated only antenatal and consultations for routine growth and development monitoring during the children’s first 2 year. Moreover, increasing provision of care may not necessarily mean increasing quality of care. Fifth, we used a cross sectional approach to perform our estimates, and further investigation should focus on causality. Fifth, we used a cross sectional approach to perform our estimates, and further investigation should focus on causality.”

Regarding the comment “Results: In the tables, please limit results to only 1 decimal point in Table 1 and no more than three decimal places in Tables 2-4. The sheer volume of numbers make the data very difficult to interpret.”, we would like to keep three decimal places for numbers less than 1, and one decimal place for numbers greater than 1. In Tables 2-4, we used 4 decimal places for numbers less than 1, since some coefficients are very low. The main argument of the study is that there are asymmetrical effects across PHT. We used quantile regression approach for this reason. If we reduce the decimal places, these asymmetrical effects will disappear, since the coefficients are very low.
